# Association of COVID-19 Mitigation Measures With Changes in Cardiorespiratory Fitness and Body Mass Index Among Children Aged 7 to 10 Years in Austria

**DOI:** 10.1001/jamanetworkopen.2021.21675

**Published:** 2021-08-26

**Authors:** Gerald Jarnig, Johannes Jaunig, Mireille N. M. van Poppel

**Affiliations:** 1 Institute of Human Movement Science, Sport and Health, University of Graz, Graz, Austria

## Abstract

**Importance:**

Previous studies have shown reductions in self-reported physical activity levels in children associated with implementation of COVID-19 mitigation measures, and data on objectively assessed health parameters are limited.

**Objective:**

To examine the association of COVID-19 mitigation measures with changes in cardiorespiratory fitness (CRF) measures and body mass index (BMI) among primary schoolchildren.

**Design, Setting, and Participants:**

This cohort study included children aged 7 to 10 years from 12 randomly selected primary schools in urban and rural districts of Klagenfurt, Austria. Baseline CRF and BMI measurements were obtained in September 2019 before COVID-19 mitigation measures were implemented, and follow-up measurements were obtained in June and September 2020.

**Exposures:**

COVID-19 mitigation measures.

**Main Outcomes and Measures:**

Cardiorespiratory fitness was measured with a 6-minute endurance run test. Height and weight were objectively measured. Standard deviation scores were calculated for CRF and BMI. Changes over time were analyzed using analyses of variance. Secondary analyses were performed for subgroups stratified by sex.

**Results:**

A total of 764 children (383 girls [50.1%]) aged 7 to 10 years had all measurements completed. From September 2019 to September 2020, CRF SD scores changed by −1.06 (95% CI, −1.13 to −1.00), with a similar decrease in both boys and girls. Body mass index SD scores had increased by 0.12 (95% CI, 0.06-0.16) in June 2020 and by 0.16 (95% CI, 0.12-0.20) in September 2020 compared with September 2019. The increase in BMI SD scores (from September 2019 to September 2020) was greater among boys (0.23; 95% CI, 0.18-0.29) than among girls (0.09; 95% CI, 0.04-0.15). During the 1-year period, the percentage of children with overweight or obesity increased from 20.3% (155 children) to 24.1% (184 children) (difference, 3.8% [29 children]).

**Conclusions and Relevance:**

In this cohort study of children in Austria, COVID-19 mitigation measures were associated with decreases in CRF measures and increases in BMI. The findings suggest that collaborative efforts are needed to reverse these changes in children’s health to prevent long-term negative health outcomes.

## Key Points

**Question:**

Were COVID-19 mitigation measures associated with changes in cardiorespiratory fitness measures and body mass index among primary schoolchildren in Austria?

**Findings:**

In this cohort study of 764 primary schoolchildren aged 7 to 10 years, COVID-19 mitigation measures were associated with substantial reductions in cardiorespiratory fitness measures and increases in body mass index SD scores and the proportion of children with overweight or obesity.

**Meaning:**

The findings suggest that collaborative efforts are needed to improve children’s health and fitness to prevent long-term negative health outcomes.

## Teaser

This cohort study examines the association of COVID-19 mitigation measures with changes in cardiorespiratory fitness measures and body mass index among primary schoolchildren aged 7 to 10 years in Austria.

## Introduction

The indirect consequences of the COVID-19 pandemic are of concern, especially the consequences for children. Studies^1,2,3,4,5,6^ worldwide have described a negative association of pandemic mitigation measures with self- or proxy-reported levels of physical activity and sedentary behavior among youths. The reported reduction in physical activity levels and the increase in sedentary behavior may be associated with changes in relevant health-related parameters, such as cardiorespiratory fitness (CRF), and indirectly with changes in body mass index (BMI).

Cardiorespiratory fitness in childhood is an important health marker,^7^ and a higher level of CRF is associated with lower measurements of BMI, waist circumference, and body fat and a reduced prevalence of metabolic syndrome in later life.^8^ Childhood obesity is associated with increased cardiovascular risk factors^9^ and coronary heart disease.^10^ However, to our knowledge, there are currently no studies on the associations of COVID-19 mitigation measures with objectively measured CRF and BMI in a representative sample of children.

As in many other countries, children in Austria had limited access to exercise and sports from March 2020 to September 2020 because playgrounds and sports facilities were closed in the spring of 2020 and children were unable to attend physical education (PE) classes in school until September 2020 (eFigure 1 in the Supplement). Therefore, we aimed to examine the associations of COVID-19 mitigation measures with changes in CRF and BMI (as continuous and dichotomized variables) among primary schoolchildren aged 7 to 10 years in Klagenfurt, Austria, from September 2019 to September 2020.

## Methods

This cohort study was originally designed as a randomized clinical trial to evaluate the effects of a PE intervention on motor competence, CRF, and health of primary schoolchildren. Because of COVID-19 regulations, the intervention had to be stopped in March 2020. The study was approved by the research ethics committee at the University of Graz, Styria, Austria. Written informed consent was obtained from legal guardians of the participating children. This study followed Strengthening the Reporting of Observational Studies in Epidemiology (STROBE) reporting guideline.^11^

### Selection of Schools and Participants

**Figure 1.**
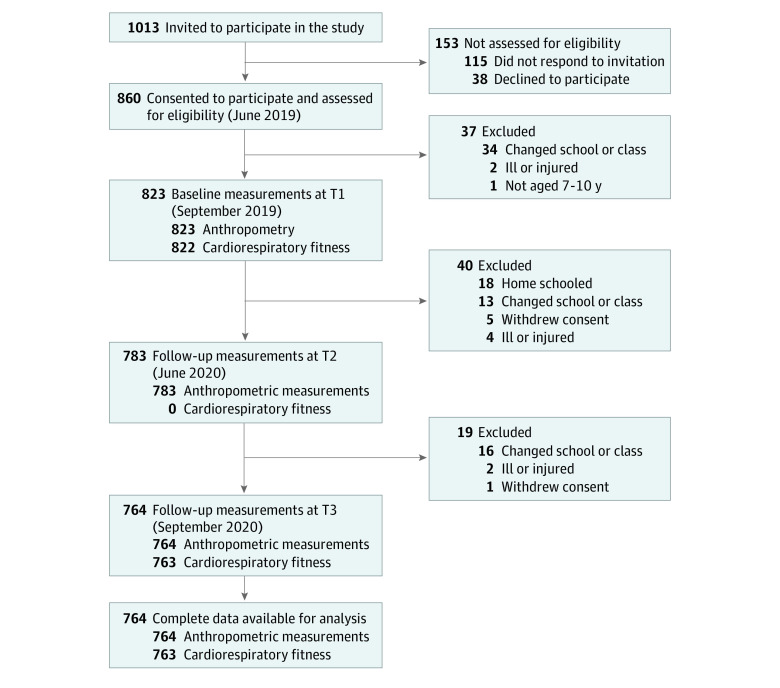
Flow Diagram T indicates time.

A list of all 39 primary schools in both urban and rural districts of Klagenfurt, Austria, was used for the selection of schools. Using a random number generator, 12 schools were selected and randomized to intervention and control groups stratified by district. Administrators of all schools consented to participate in the study. Inclusion criteria were age of 7 to 10 years at baseline and the physical ability to perform all motor competence tests in the test battery. Between May 1 and June 28, 2019, we invited all 1013 children attending these 12 schools to participate. Legal guardians of 860 (85%) children provided written informed consent for their children’s participation (Figure 1).

### Procedures

Baseline measurements were performed in September and October 2019 (T1). When the lockdown restrictions were slowly relaxed (eFigure 1 in the Supplement), the legal department of the Education Directorate of Carinthia allowed a second test phase in May and June 2020 (T2) with the strict hygiene measures and regulations applicable at that time.^12^ The CRF test was not permitted at that time because the minimum distance between participants could not be ensured. In September and October 2020 (T3), the third test phase was conducted, during which the complete test battery was performed.

The intervention was started after baseline measurements were obtained in October 2019. In the intervention group, external trainers and sports scientists planned and taught all PE classes. The intervention and usual PE classes (control group) were interrupted by the lockdown on March 16, 2020.^12^

The Oxford COVID-19 Government Response Tracker provided internationally comparable stringency levels for Austria during the study period (eFigure 1 in the Supplement); a more detailed self-developed stringency level of the pandemic mitigation measures for children over time is given in eFigure 1 in the Supplement. The precise description of this classification method, based on the Austrian legislative decrees accessible in the federal law gazette,^12^ is given in eTable 1 in the Supplement.

### Outcomes

In this study, the primary outcomes were changes in CRF and BMI. Secondary analyses were performed for subgroups stratified by sex and sports club membership. Whether children were members of a sports club was reported by legal guardians in the baseline questionnaire. The anthropometric data included height in centimeters and weight in kilograms. Height was measured to the nearest 0.1 cm with a portable stadiometer (seca 213). Weight was measured to the nearest 0.1 kg using an electronic weight scale (Bosch PPW4202). Each participant’s BMI was calculated as weight in kilograms divided by height in meters squared. As a measure of CRF, we used the 6-minute endurance run test (hereafter referred to as *6-minute run*)^13,14^ because this is the most relevant fitness parameter for future cardiovascular health status.^8^ The 6-minute run was performed according to the protocol of the Düsseldorfer Modell.^13^

#### Standardization of the 6-Minute Run

From the results of the 6-minute run (raw score), SD scores were created on the basis of age- and sex-specific reference values. Because no reference values were available for children at this age in Austria, we used German references from the Düsseldorfer Modell (collected from 2011 to 2018),^13^ which are based on the LMS method.^15^ We used reference values from the German Motor Test^14^ in sensitivity analyses to check the robustness of the findings (eMethods in the Supplement).

#### Standardization of BMI

Age- and sex-specific reference values were obtained from the International Obesity Taskforce,^16^ and SD scores for BMI were calculated according to the LMS method.^15^ The International Obesity Taskforce reference uses a centile curve approach^16^ with values corresponding to BMI at the age of 18 years. This approach allows BMI thresholds to be on a continuum with those used for adults (ie, overweight defined as BMI≥25). In addition, BMI was dichotomized using thresholds for below and above the definition of overweight. We used alternative national^17,18^ and international^19^ reference values in sensitivity analyses to check the robustness of the findings (eMethods in the Supplement).

### Statistical Analysis

Descriptive statistics were calculated for all 3 time points. Continuous variables are expressed as means (SDs), and categorial variables are expressed as absolute values and percentages. The analyzed data only include complete data for all measured time points (Figure 1), and no data imputation was performed.

#### Changes Over Time

In multilevel mixed models with individual and school as the 2 random levels, possible clustering of outcomes within schools (n = 12) and the association of possible confounders (age, allocation to intervention, and residential area [rural or urban]) with the outcomes were assessed. Because we observed no significant clustering and no association of possible confounders with the outcomes, further analyses were performed using 3-way analysis of variance with repeated measurements. Sex and sports club membership (yes or no) were entered into the models as between-participant effects, and the measurement time points (3 for BMI and 2 for 6-minute run) were entered as within-participant effects. In cases of nonsphericity, the Greenhouse-Geisser correction was performed. Homogeneity was tested with the Hartley Fmax test. ^20^ To visualize interaction effects, the scores in the figures were plotted by sex and by sports club membership on separate lines, with statistics for significant main effects and interaction effects described within.

Differences in the dichotomized BMI classification (underweight and normal weight vs overweight and obese) were analyzed for the total sample and for the subgroups (sex and sports club membership) using the Cochran Q test for a possible time effect. The Dunn test was subsequently used to investigate the effects between the pairwise measurement time points (T1 to T2, T1 to T3, and T2 to T3).

All tests were 2-sided, with *P* < .05 considered statistically significant. The α level correction for post hoc tests was performed using Bonferroni correction. For analysis of variance, partial η^2^ (η_p_^2^) was used to determine the size of the effect (≥0.01, small; ≥0.06, medium; ≥0.14, large); thus, small effects and larger were considered relevant. All statistical calculations were performed using SPSS, version 26 (IBM Corp).

## Results

In September 2019, baseline measurements were completed for 823 children. Fifty-nine children (7%) did not have complete assessments at all 3 measurement time points and were excluded from analyses, resulting in 764 children with complete anthropometric data; 60 children (7%) did not have baseline measurements of CRF and were also excluded from analyses, resulting in 763 children with complete data for CRF (Figure 1). The included study population and the group lost to follow-up were compared in terms of age, sex, sports club membership, region, BMI, and CRF. Between-group comparisons revealed only a difference in the 6-minute run (eTables 2 and 3 in the Supplement). Characteristics of the sample overall and by sex are shown in Table 1 and eTable 4 in the Supplement. In the study population, the mean (SD) age at baseline was 8.3 (0.7) years, 383 (50.1%) participants were girls, 322 (42.1%) were members of a sports club, and 451 (59.0%) lived in the urban region of Klagenfurt.

**Table 1.** Characteristics for the Sample Overall and by Sex and Sports Club Membership

Characteristic	September 2019	June 2020	September 2020
**6-min Run, mean (SD), m**			
All (n = 763)	917.0 (141.0)	ND	815.0 (134.3)
Sports club membership	966.8 (131.9)	ND	860.0 (135.6)
No sports club membership	880.9 (136.5)	ND	782.2 (123.4)
Girls			
All	871.2 (121.4)	ND	777.4 (118.7)
Sports club membership	900.9 (113.4)	ND	797.0 (127.8)
No sports club membership	857.8 (122.8)	ND	768.6 (113.6)
Boys			
All	963.1 (144.5)	ND	852.8 (138.4)
Sports club membership	1005.5 (126.7)	ND	897.0 (126.4)
No sports club membership	915.0 (148.6)	ND	802.4 (134.6)
**6-min Run SD score, mean (SD)**			
All (n = 763)	0.49 (1.13)	ND	−0.57 (0.97)
Sports club membership	0.83 (1.05)	ND	−0.29 (0.97)
No sports club membership	0.24 (1.12)	ND	−0.78 (0.92)
Girls			
All	0.40 (1.08)	ND	−0.64 (0.96)
Sports club membership	0.67 (1.00)	ND	−0.48 (1.02)
No sports club membership	0.28 (1.09)	ND	−0.71 (0.92)
Boys			
All	0.58 (1.17)	ND	−0.50 (0.98)
Sports club membership	0.93 (1.06)	ND	−0.18 (0.92)
No sports club membership	0.19 (1.17)	ND	−0.88 (0.91)
**BMI SD score, mean (SD)**			
All (n = 764)	0.37 (1.08)	0.49 (1.10)	0.53 (1.10)
Sports club membership	0.26 (0.96)	0.36 (1.01)	0.40 (1.00)
No sports club membership	0.44 (1.16)	0.59 (1.15)	0.63 (1.16)
Girls			
All	0.45 (1.08)	0.54 (1.10)	0.56 (1.13)
Sports club membership	0.27 (0.96)	0.32 (1.00)	0.35 (1.06)
No sports club membership	0.54 (1.13)	0.64 (1.13)	0.65 (1.15)
Boys			
All	0.28 (1.08)	0.45 (1.10)	0.50 (1.07)
Sports club membership	0.25 (0.96)	0.38 (1.02)	0.43 (0.97)
No sports club membership	0.30 (1.20)	0.52 (1.18)	0.59 (1.17)
**Overweight and obesity, No. (%)** ^a^			
Total (n = 764)	155 (20.3)	169 (22.1)	184 (24.1)
Sports club membership (n = 322)	51 (15.8)	58 (18.0)	65 (20.2)
No sports club membership (n = 442)	104 (23.5)	111 (25.1)	119 (26.9)
Girls			
Total (n = 383)	91 (23.8)	97 (25.3)	103 (26.9)
Sports club membership (n = 119)	20 (16.8)	24 (20.2)	26 (21.8)
No sports club membership (n = 264)	71 (26.9)	73 (27.7)	77 (29.2)
Boys			
Total (n = 381)	64 (16.8)	72 (18.9)	81 (21.3)
Sports club membership (n = 203)	31 (15.3)	34 (16.7)	39 (19.2)
No sports club membership (n = 178)	33 (18.5)	38 (21.3)	42 (23.6)

Abbreviations: 6-min run, 6-minute run endurance test; BMI, body mass index (calculated as weight in kilograms divided by height in meters squared); ND, not determined.

^a^ Defined as a BMI of 25 or greater.

### Change in CRF

**Figure 2.**
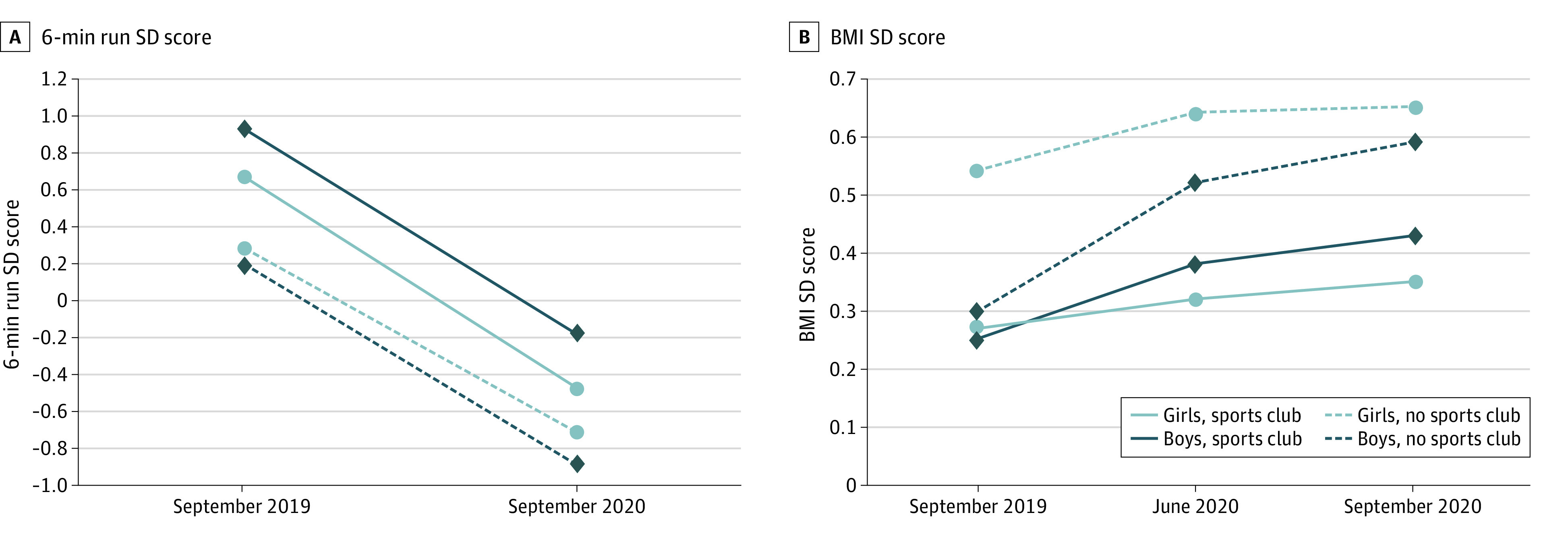
Changes in Cardiorespiratory Fitness (CRF) Measures and Body Mass Index (BMI) by Subgroup Between September 2019 and September 2020 CRF was assessed with the 6-minute endurance run test (6-min run). From the results of the 6-minute run (raw score), SD scores were created on the basis of age- and sex-specific reference values. BMI was calculated as weight in kilograms divided by height in meters squared. SD scores for BMI were calculated according to the LMS method.^15^

From September 2019 to September 2020, the mean (SD) distance that the children were able to run in 6 minutes decreased from 917.0 (141.1) meters to 815.0 (134.3) meters. The mean (SD) 6-minute run SD score decreased from 0.49 (1.13) to −0.57 (0.97); CRF SD scores changed by −1.06 (95% CI, −1.13 to −1.00), with a similar decrease in both boys and girls (η_p_^2^ = 0.554; *P* < .001). Although children who were members of sports clubs had better CRF measures at all time points, the decrease in CRF, as assessed by the 6-minute run over time, was similar in all groups (Figure 2A). Table 2 gives detailed results of the analyses of variance (eTable 5 in the Supplement shows results of sensitivity analyses).

**Table 2.** Three-Way Mixed Analyses of Variance for 6-Minute Endurance Run Test and Body Mass Index SD Scores

Variable	*df*	*F*	*P* value	η_p_^2^	Power^a^
**6-min Run SD score**					
Between-participants effects					
Sex	1	1.39	.24	.002	.22
Sports club membership	1	58.20	<.001	.071	>.99
Sex × sports club	1	9.21	.002	.012	.86
Error	759	NA	NA	NA	NA
Within-participant effects					
Time: T1 − T3	1	943.77	<.001	.554	>.99
Time × sex	1	0.06	.80	<.001	.06
Time × sports club membership	1	1.75	.19	.002	.26
Time × sex × sports club membership	1	0.74	.39	.001	.14
Error: time	759	NA	NA	NA	NA
**BMI SD score**					
Between-participants effects					
Sex	1	0.37	.54	<.001	.09
Sports club membership	1	6.68	.01	.009	.73
Sex × sports club membership	1	1.22	.27	.002	.20
Error	760	NA	NA	NA	NA
Within-participant effects					
Time: T1 − T2 − T3	1.96	56.84	<.001	.070	>.99
Time × sex	1.96	.05	<.001	.013	.98
Time × sports club membership	1.96	3.92	.02	.005	.70
Time × sex × sports club	1.96	0.73	.48	.001	.17
Error: time	1489.79	NA	NA	NA	NA

Abbreviations: 6-min run, 6-minute run endurance test; ANOVA, analysis of variance; BMI, body mass index; NA, not applicable; η_p_^2^, partial η^2^.

^a^ Observed power computed using α  = .05.

### Change in BMI

We observed a significant increase in the BMI SD score over time (η_p_^2^ = 0.070; *P* < .001). The mean (SD) BMI SD score was 0.37 (1.08) in September 2019 and 0.49 (1.10) in June 2020; the difference in scores was more pronounced than that between June 2020 and September 2020, when the mean (SD) BMI SD score was 0.53 (1.10). Body mass index SD scores had increased by 0.12 (95% CI, 0.06-0.16) in June 2020 and by 0.16 (95% CI, 0.12-0.20) in September 2020 compared with September 2019. Means (SDs) for all subgroups by time point are presented in Table 1. Boys had a greater change in BMI SD score over time than girls (boys: 0.23 [95% CI, 0.18-0.29]; girls: 0.09 [95% CI, 0.04-0.15]; time × sex: η_p_^2^ = 0.013; *P* < .001). Changes over time are shown in Figure 2B and Table 2. Post hoc tests for analyses of variance are presented in eTable 6 in the Supplement. Similar changes were found in the sensitivity analyses for Austrian and World Health Organization reference values (eTables 6 and 7 in the Supplement).

**Figure 3.**
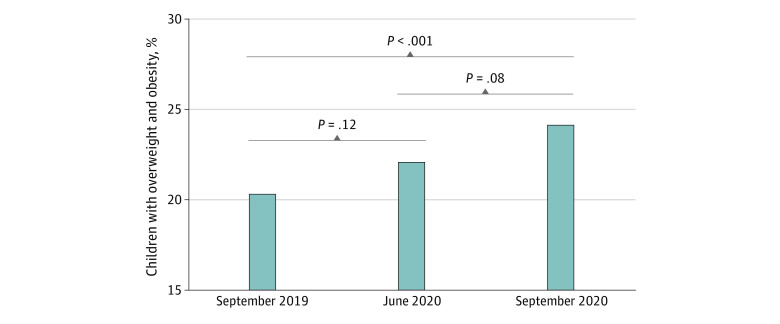
Percentage of Children With Overweight and Obesity According to International Obesity Taskforce Thresholds Bonferroni corrected pairwise comparisons were performed between the measurement time points. Overweight and obesity was defined as a body mass index of 25 or greater (calculated as weight in kilograms divided by height in meters squared).

The increase in the BMI SD score was associated with an increased proportion of children classified as having overweight or obesity. In September 2019, 91 girls (23.8%) and 64 boys (16.8%) had overweight or obesity (Table 1). The proportion of children who had overweight or obesity had increased by 1.8% between September 2019 and June 2020 (from 155 [20.3%] to 169 [22.1%] children), with increases of 1.5% among girls (from 91 [23.8%] to 97 [25.3%] girls) and 2.1% among boys (from 64 [16.8%] to 72 [18.9%] boys), and by 3.8% between September 2019 and September 2020 (184 [24.1%]), with increased of 3.1% among girls (103 [26.9%]) and 4.5% among boys (81 [21.3%]) (Table 1, Figure 3). In the subgroups, different patterns of changes in the proportion of children with overweight or obesity were observed (Table 1 and eFigure 2 in the Supplement). Statistical tests for dichotomized BMIs in the total sample and subgroups are shown in eTables 8 to 11 in the Supplement. Although the proportions of children with overweight or obesity were different when Austrian and World Health Organization thresholds were used, the increase from September 2019 to September 2020 was significant for all 3 methods used to categorize BMI (eTables 8-12 in the Supplement).

## Discussion

To our knowledge, this study is the first to address the association of COVID-19 mitigation measures with objective health-related parameters in a representative sample of primary schoolchildren. We observed a reduction in CRF measures and an increase in BMI from September 2019 to September 2020.

Decreased CRF measures may be associated with a reduction in physical activity,^7,8,9,10^ especially a reduction in activities of higher intensity, such as those performed in physical education classes and during sports activities. These findings are in line with the results from a study with a small sample size that reported decreased CRF measures in 10 children during a COVID-19–related lockdown compared with 10 matched control participants from before the lockdown in the US.^21^ A study of 25 adolescent elite soccer players reported a 9% reduction in oxygen consumption as determined by aerobic capacity testing after 8 weeks of home confinement^22^ despite participation in a home training program. The magnitude of the decrease in CRF measures in the present study (effect size for the 6-minute run) was approximately 1 SD in all subgroups stratified by sex and sports club membership, which could be qualified as a large effect (η_p_^2^ = 0.554).

The proportion of children with overweight or obesity increased by 3.1% among girls and 4.5% among boys. Although a reduction in energy expenditure owing to lower physical activity levels may have been a contributing factor, the increase in BMI SD score was likely associated with a combination of various factors that changed during the COVID-19 pandemic. In children, changes in diet,^5^ mental health, and increased sedentary time have been reported, all of which may have been associated with an increase in BMI.^23,24,25^ The 3.8% increase in the number of children with overweight or obesity in the 1-year period is similar in magnitude to the increase seen in a 4-year period (2014 to 2018) in Austrian children.^26^ Another longitudinal study investigating primary schoolchildren from the wider Bristol, UK, area over a 4-year period (2012 to 2016) reported a 5.7% increase in overweight and obesity.^27^ Another study reporting data on the association of the COVID-19 mitigation measures with objectively measured BMI reported a mean increase of 0.22 in BMI SD score in Korean children,^28^ which is higher than the increase we observed in the present study. This difference might be attributable to the use of different methods selection of the samples (specific reasons for clinic visit [Korea] vs random selection of primary schoolchildren [Austria]) or stricter mitigation measures in Korea.^29^

### Implications

The observed changes in CRF measures and BMI may be transitory because children will likely recover from the restrictions imposed on their lives. However, this might not be true for all children. To our knowledge, there is no information on the recovery of healthy children after a long period of enforced inactivity. The results presented here are the changes that occurred from September 2019 to September 2020, and since then, children in Austria have again experienced partial lockdown, which will likely have additional implications for their health. In the meantime, interventions to ensure that children recover to an age-adequate level of CRF and BMI may be needed. This would mean investing time and effort in physical education classes in schools and encouraging children to be active during their leisure time. We believe that schools should focus not only on addressing the deficits in academic learning associated with COVID-19 measures, which is unarguably important, but also on physical development of children. In addition to COVID-19, other prevailing pandemics of nontransmissible diseases related to obesity and lack of physical activity should be considered.^24,30^

### Strengths and Limitations

This study has strengths. The study population was representative of children in the whole region of Klagenfurt, including both rural and urban areas. Participation rates were high, and loss to follow-up was low and nonselective. A between-group comparison of the study population and the group lost to follow-up showed a difference only in the 6-minute run performance. The reasons for dropping out of the study were primarily a change in school, COVID-19–related homeschooling, or illness or injury; only 6 children left the study because consent was withdrawn (Figure 1). Therefore, our results may be generalized to all parts of Austria where identical COVID-19 mitigation measures were implemented and possibly to other countries with similar mitigation measures. The objective, longitudinal measurements of weight, height, and CRF were unique because all previous studies assessing the association of COVID-19 mitigation measures with health parameters of children used self-reported data. The 2 measurements were exactly a year apart (September 2019 and September 2020), ruling out seasonal influences on CRF and BMI values.

This study also has limitations. We did not design the study for the purpose of the analyses presented in this article. We did not collect data on factors that could have been associated with BMI or CRF, such as diet, sleep, physical activity, or mental health. We also did not have a control group of children unaffected by the COVID-19 mitigation measures; thus, causal inference cannot be made. Because of the lack of a control group and because both the 6-minute run performance and the BMI measurements in growing children change over time, we compared the results from the study sample with several established age- and sex-specific references as a control. With this approach, SD scores and values for CRF and BMI would be expected to remain stable over time, even when the raw scores of the 6-minute run and the anthropometric measurements changed over time. However, we observed significant changes in SD scores, suggesting a change associated with reference cohorts. Given the magnitude of changes in CRF measures and BMI, the changes were unlikely attributable to natural variations. Regardless of which reference values were used,^13,14^ the decrease in CRF measures remained the same. Results from sensitivity analyses for BMI SD scores using national and international reference values showed similar, significant increases in BMI SD scores over time. The only differences were found in BMI categories when international^16,19^ or Austrian^17^ reference values were used.

## Conclusions

In this cohort study of Austrian children aged 7 to 10 years, CRF levels decreased and BMI increased from September 2019 to September 2020, most likely in association with the COVID-19 mitigation measures. The findings suggest that efforts should be made to improve these health parameters, which are relevant for the long-term health of children.

## Notes

Supplement.
eMethods.
**eTable 1.** Restriction Levels for Children in Austria From January 31, 2020 to September 30, 2020 in Relation to the OxCGRT Stringency Index **eTable 2.** Overall Sample Characteristics Study Population vs Loss at Follow-up **eTable 3.** Detailed Sample Characteristics Study Population vs Loss at Follow-up **eTable 4.** Additional Sample Characteristics for Boys and Girls by Sports Club Membership **eTable 5.** 3-Way Mixed ANOVAs for 6-min Run SDS Using GMT Reference Values **eTable 6.** Post-hoc Tests for BMI SDS for the Main Effect Time and Time*Gender Interaction Based on the Estimated Marginal Means **eTable 7.** 3-Way Mixed ANOVAs for BMI SDS Using Austrian and WHO Reference Values **eTable 8.** Cochran's Q Test for Dichotomous BMI Classification Using IOTF, Austrian and WHO Reference Values **eTable 9.** Post-hoc Tests for Dichotomous BMI Classification Using IOTF, Austrian and WHO Reference Values **eTable 10.** Cochran's Q Test for Dichotomous BMI Classification Using IOTF, Austrian and WHO Reference Values **eTable 11.** Post-hoc Tests for Dichotomous BMI Classification Using IOTF, Austrian and WHO Reference Values **eTable 12.** Children with Overweight and Obesity Using Austrian and WHO Reference Thresholds **eFigure 1.** COVID-19 Restrictions in Austria Between January 31, 2020 and September 30, 2020 **eFigure 2.** Percentage of Overweight and Obese Children According to IOTF Thresholds for Girls and Boys by Sports Club Membership
