# Supplementary material for: Association of COVID-19 Mitigation Measures With Changes in Cardiorespiratory Fitness and Body Mass Index Among Children Aged 7 to 10 Years in Austria
Source: JAMA Netw Open. 2021 Aug 26;4(8):e2121675. doi: 10.1001/jamanetworkopen.2021.21675 (PMC8391099; doi:10.1001/jamanetworkopen.2021.21675)
Supplement: Supplement. — eMethods. eTable 1. Restriction Levels for Children in Austria From January 31, 2020 to September 30, 2020 in Relation to the OxCGRT Stringency Index eTable 2. Overall Sample Characteristics Study Population vs Loss at Follow-up eTable 3. Detailed Sample Characteristics Study Population vs Loss at Follow-up eTable 4. Additional Sample Characteristics for Boys and Girls by Sports Club Membership eTable 5. 3-Way Mixed ANOVAs for 6-min Run SDS Using GMT Reference Values eTable 6. Post-hoc Tests for BMI SDS for the Main Effect Time and Time*Gender Interaction Based on the Estimated Marginal Means eTable 7. 3-Way Mixed ANOVAs for BMI SDS Using Austrian and WHO Reference Values eTable 8. Cochran's Q Test for Dichotomous BMI Classification Using IOTF, Austrian and WHO Reference Values eTable 9. Post-hoc Tests for Dichotomous BMI Classification Using IOTF, Austrian and WHO Reference Values eTable 10. Cochran's Q Test for Dichotomous BMI Classification Using IOTF, Austrian and WHO Reference Values eTable 11. Post-hoc Tests for Dichotomous BMI Classification Using IOTF, Austrian and WHO Reference Values eTable 12. Children with Overweight and Obesity Using Austrian and WHO Reference Thresholds eFigure 1. COVID-19 Restrictions in Austria Between January 31, 2020 and September 30, 2020 eFigure 2. Percentage of Overweight and Obese Children According to IOTF Thresholds for Girls and Boys by Sports Club Membership [file jamanetwopen-e2121675-s001.pdf]

Supplementary Online Content

Jarnig G, Jaunig J, van Poppel MNM. Association of COVID-19 mitigation measures with changes in cardiorespiratory fitness and body mass index among children aged 7 to 10 years in Austria. *JAMA Netw Open*. 2021;4(8):e2121675. doi:10.1001/jamanetworkopen.2021.21675

eMethods.

eTable 1. Restriction Levels for Children in Austria From January 31, 2020 to September 30, 2020 in Relation to the OxCGRT Stringency Index

eTable 2. Overall Sample Characteristics Study Population vs Loss at Follow-up

eTable 3. Detailed Sample Characteristics Study Population vs Loss at Follow-up

eTable 4. Additional Sample Characteristics for Boys and Girls by Sports Club Membership

eTable 5. 3-Way Mixed ANOVAs for 6-min Run SDS Using GMT Reference Values

eTable 6. Post-hoc Tests for BMI SDS for the Main Effect Time and Time*Gender Interaction Based on the Estimated Marginal Means

eTable 7. 3-Way Mixed ANOVAs for BMI SDS Using Austrian and WHO Reference Values

eTable 8. Cochran's Q Test for Dichotomous BMI Classification Using IOTF, Austrian and WHO Reference Values

eTable 9. Post-hoc Tests for Dichotomous BMI Classification Using IOTF, Austrian and WHO Reference Values

eTable 10. Cochran's Q Test for Dichotomous BMI Classification Using IOTF, Austrian and WHO Reference Values

eTable 11. Post-hoc Tests for Dichotomous BMI Classification Using IOTF, Austrian and WHO Reference Values

eTable 12. Children with Overweight and Obesity Using Austrian and WHO Reference Thresholds

eFigure 1. COVID-19 Restrictions in Austria Between January 31, 2020 and September 30, 2020

eFigure 2. Percentage of Overweight and Obese Children According to IOTF Thresholds for Girls and Boys by Sports Club Membership

This supplementary material has been provided by the authors to give readers additional information about their work.

© 2021 Jarnig G et al. *JAMA Network Open*.

This supplementary material has been provided by the authors to give readers additional information about their work.

eMethods

To check for robustness of the main outcomes, sensitivity analyses were conducted. Thus, alternative references values were used for cardiorespiratory fitness (CRF) and body mass index (BMI). The results are presented in the following eTables.

Standardization of the 6-min run

Based on age- and gender-specific references from the German Motor Test (GMT),[14] standard deviation scores (SDS) were calculated using traditional z-score standardisation (referred to as 6-min run SDS [GMT]).

Standardization of the body mass index

For national reference values, recent data from a large representative Austrian sample were used.[17,18] The Austrian reference data were calculated using GAMLSS models (see Gleiss et al[18] and Mayer et al[17] for detailed statistical procedure). National reference values are expressed in BMI centile curves (ie, equicurves), representing BMI values corresponding to values at the age of 18 years (hereinafter referred to as EQUI BMI [AUT]). This approach allows BMI thresholds to be on a continuum to those used for adults (ie, overweight BMI $\geq$ 25 kg/m²).
For comparison with alternative international reference values, the WHO[19] references were considered, whereas the calculations were based on the LMS method.[20] The WHO reference values are expressed in SDS (referred to as BMI SDS [WHO]), but it uses +1 SDS as a threshold for overweight.[19] Therefore, the dichotomous classification results (underweight and normal weight vs. overweight and obesity) of the particular references were calculated using their given thresholds, respectively.

© 2021 Jarnig G et al. *JAMA Network Open*.

eTable 1. Restriction levels for children in Austria from January 31, 2020 to September 30, 2020 in relation to the OxCGRT stringency index

**Period**	**OxCGRT Austria**	**Sschool structure**	**Physical education in school**	**Sports and leisure facilities**	**Sport at the club**	**Stay at home requirements**
01-31-2020 – 02-23-2020	0	0	0	0	0	0
02-24-2020 – 03-08-2020	11.11	0	0	0	0	0
03-09-2020 – 03-11-2020	19.44	0	0	0	0	0
03-12-2020	34.26	0	0	0	0	0
03-13-2020 – 03-15-2020	48.15	0	0	0	0	0
03-16-2020 – 04-13-2020	81.48	3	3	3	3	3
04-14-2020 – 04-22-2020	77.78	3	3	3	3	3
04-23-2020 – 04-30-2020	75.00	3	3	3	3	3
05-01-2020	67.59	3	3	1	3	1
05-02-2020 – 05-10-2020	64.81	3	3	1	3	1
05-11-2020 – 05-17-2020	59.26	3	3	1	3	1
05-18-2020 – 05-28-2020	59.26	2	3	1	3	1
05-29-2020 – 06-02-2020	53.70	2	3	1	2	1
06-03-2020	50.00	2	3	1	2	1
06-04-2020 – 07-01-2020	47.22	2	3	1	2	1
07-02-2020 – 07-09-2020	47.22	2	3	0	0	1
07-10-2020 – 09-05-2020	35.19	0	0	0	0	1
09-06-2020 – 09-13-2020	36.11	0	0	0	0	1
09-14-2020 – 09-16-2020	36.11	0	0	0	0	1
09-17-2020 – 09-28-2020	37.04	0	0	0	0	1
09-29-2020 – 09-30-2020	40.74	0	0	0	0	1

Level 0 = no restrictions or holidays, Level 1 = low restrictions, Level 2 = medium restrictions, Level 3 = high restrictions, OxCGRT = Oxford COVID-19 Government Response Tracker Stringency Index

© 2021 Jarnig G et al. *JAMA Network Open*.

eTable 2. Overall sample characteristics study population vs. loss at follow-up

	Study population	loss at follow-up	X^2	t	*P* value
**Age (years)**	8.3 (0.7)	8.1 (0.7)		1.22	.22
**Female gender**	383 (50.1%)	36 (61.0%)	2.597		.11
**Member of sports club**	322 (42.1%)	27 (45.8%)	0.293		.59
**Urban region**	451 (59%)	34 (58%)	0.045		.83
**BMI SDS**	0.37 (1.08)	0.28 (1.13)		0.57	.57
**BMI categories (ow+ob)**	155 (20.3%)	12 (20.3%)	0.000		.99
**6-min run (m)**	917.0 (141.0)	854.1 (117.4)		3.34	< .001
**6-min run SDS**	0.49 (1.13)	0.02 (0.98)		3.09	.002

Sample size for study population, N = 764 (6 min run = 763); for loss at follow up, N = 59.
Data are n (%) or mean (SD). DüMo = 6-min run SDS based on the Düsseldorfer Modell (Stemper et al, 2020), IOTF = BMI SDS based on International Obesity Taskforce reference centile curves (Cole et al, 2012), m = meter, SDS = standard deviation score.
X^2= Chi-Square Test value; t=test statistic t-test

© 2021 Jarnig G et al. *JAMA Network Open*.

eTable 3. Detailed Sample characteristics study population vs. loss at follow up

			Study population		loss at follow-up	
**Weight (kg)**	sports club total		n=322	29.1 (6.1)	n=27	28.6 (7.1)
	no sports club total		n=442	30.4 (7.9)	n=32	28.6 (7.2)
	girls	total	n=383	29.9 (7.1)	n=36	27.1 (6.2)
		sports club	n=119	28.7 (6.1)	n=17	26.3 (4.2)
		no sports club	n=264	30.4 (7.4)	n=19	27.8 (7.6)
	boys	total	n=381	29.8 (7.3)	n=23	30.9 (7.9)
		sports club	n=203	29.3 (6.1)	n=10	32.4 (9.3)
		no sports club	n=178	30.3 (8.5)	n=13	29.7 (6.7)
**Height (cm)**	sports club total		n=322	132.2 (6.5)	n=27	129.9 (6.2)
	no sports club total		n=442	132.3 (6.7)	n=32	130.5 (8.9)
	girls	total	n=383	131.7 (6.8)	n=36	129.8 (7.3)
		sports club	n=119	131.3 (6.6)	n=17	130.0 (5.8)
		no sports club	n=264	131.9 (6.9)	n=19	130.6 (8.4)
	boys	total	n=381	132.7 (6.4)	n=23	130.9 (6.4)
		sports club	n=203	132.7 (6.3)	n=10	131.5 (6.8)
		no sports club	n=178	132.8 (6.5)	n=13	130.5 (6.4)
**BMI SDS**	sports club total		n=322	0.26 (0.96)	n=27	0.36 (1.05)
	no sports club total		n=442	0.44 (1.16)	n=32	0.22 (1.21)
	girls	total	n=383	0.45 (1.08)	n=36	-0.02 (1.02)
		sports club	n=119	0.27 (0.96)	n=17	-0.00 (0.82)
		no sports club	n=264	0.54 (1.13)	n=19	-0.05 (1.19)
	boys	total	n=381	0.28 (1.08)	n=23	0.76 (1.15)
		sports club	n=203	0.25 (0.96)	n=10	0.96 (1.15)
		no sports club	n=178	0.30 (1.20)	n=13	0.61 (1.16)
**BMI categories (ow+ob)**	sports club total		n=322	51 (15.8%)	n=27	5 (18.5%)
	no sports club total		n=442	104 (23.5%)	n=32	7 (21.9%)
	girls	total	n=383	91 (23.8%)	n=36	4 (11.1%)
		sports club	n=119	20 (16.8%)	n=17	1 (5.9%)
		no sports club	n=264	71 (26.9%)	n=19	3 (15.8%)
	boys	total	n=381	64 (16.8%)	n=23	8 (34.8%)
		sports club	n=203	31 (15.3%)	n=10	4 (40.0%)
		no sports club	n=178	33 (18.5%)	n=13	4 (30.8%)
**6-min run (m)**	sports club total		n=322	966.8 (131.9)	n=27	864.5 (124.3)
	no sports club total		n=442	880.9 (136.5)	n=32	845.3 (112.4)
	girls	total	n=383	871.2 (121.4)	n=36	837.9 (117.3)
		sports club	n=119	900.9 (113.4)	n=17	854.9 (118.0)
		no sports club	n=264	857.8 (122.8)	n=19	822.6 (117.6)
	boys	total	n=381	963.1 (144.5)	n=23	879.5 (115.5)
		sports club	n=202	1005.5 (126.7)	n=10	880.8 (139.4)
		no sports club	n=178	915.0 (148.6)	n=13	878.5 (99.3)
**6-min run SDS**	sports club total		n=322	0.83 (1.05)	n=27	0.15 (1.06)
	no sports club total		n=442	0.24 (1.12)	n=32	-0.09 (0.91)
	girls	total	n=383	0.40 (1.08)	n=36	0.13 (1.03)
		sports club	n=119	0.67 (1.00)	n=17	0.32 (1.03)
		no sports club	n=264	0.28 (1.09)	n=19	-0.05 (1.02)
	boys	total	n=381	0.58 (1.17)	n=23	-0.14 (0.89)
		sports club	n=202	0.93 (1.06)	n=10	-0.12 (1.12)
		no sports club	n=178	0.19 (1.17)	n=13	-0.14 (0.73)

Sample size for study population, N = 764 (6 min run = 763); for loss at follow up, N = 59.
Data are n (%) or mean (SD). DüMo = 6-min run SDS based on the Düsseldorfer Modell (Stemper et al, 2020), IOTF = BMI SDS based on International Obesity Taskforce reference centile curves (Cole et al, 2012), m = meter, SDS = standard deviation score.
X^2= Chi-Square Test value; t=test statistic t-test, p-lvl (p-value level) * = *P* <.05, * = *P* <.01, * = *P* <.001,

© 2021 Jarnig G et al. *JAMA Network Open*.

eTable 4. Additional sample characteristics for boys and girls by sports club membership

			September 2019	June 2020	September 2020
**Age (years)**			8.3 (0.7)	9.0 (0.7)	9.2 (0.7)
**Female gender**			383 (50.1%)		
**Member of sports club**			322 (42.1%)		
**Sports Club Membership, No. (%)**	girls	sports club	119 (15.6%)		
		no sports club	264 (34.6%)		
	boys	sports club	203 (26.6%)		
		no sports club	178 (23.3%)		
**Urban region**			451 (59%)	451 (59%)	451 (59%)
**Weight (kg)**	total		29.9 (7.2)	33.1 (8.5)	34.5 (8.9)
	sports club total		29.1 (6.1)	32.1 (7.4)	33.4 (7.7)
	no sports club total		30.4 (7.9)	33.8 (9.1)	35.2 (9.7)
	girls	total	29.9 (7.1)	33.1 (8.3)	34.5 (8.9)
		sports club	28.7 (6.1)	31.6 (7.2)	32.9 (7.8)
		no sports club	30.4 (7.4)	33.8 (8.7)	35.2 (9.3)
	boys	total	29.8 (7.3)	33.1 (8.6)	34.4 (9.0)
		sports club	29.3 (6.1)	32.4 (7.5)	33.6 (7.7)
		no sports club	30.3 (8.5)	33.9 (9.7)	35.3 (10.2)
**Height (cm)**	total		132.2 (6.6)	136.4 (6.9)	138.1 (6.9)
	sports club total		132.2 (6.5)	136.3 (6.7)	138.0 (6.8)
	no sports club total		132.3 (6.7)	136.5 (7.0)	138.2 (7.0)
	girls	total	131.7 (6.8)	136.0 (7.1)	137.8 (7.1)
		sports club	131.3 (6.6)	135.6 (6.9)	137.3 (7.1)
		no sports club	131.9 (6.9)	136.2 (7.2)	138.0 (7.2)
	boys	total	132.7 (6.4)	136.8 (6.6)	138.4 (6.6)
		sports club	132.7 (6.3)	136.7 (6.6)	138.3 (6.6)
		no sports club	132.8 (6.5)	137.0 (6.6)	138.5 (6.7)
**EQUI BMI (AUT)**	total		22.2 (3.5)	22.6 (3.8)	22.7 (3.8)
	sports club total		21.9 (2.8)	22.1 (3.1)	22.2 (3.1)
	no sports club total		22.5 (4.0)	22.9 (4.2)	23.0 (4.3)
	girls	total	22.1 (3.5)	22.3 (3.6)	22.3 (3.7)
		sports club	21.4 (2.7)	21.5 (2.9)	21.6 (3.1)
		no sports club	22.4 (3.7)	22.6 (3.8)	22.7 (3.9)
	boys	total	22.4 (3.6)	22.9 (3.9)	23.0 (4.0)
		sports club	22.2 (2.8)	22.5 (3.1)	22.6 (3.0)
		no sports club	22.6 (4.3)	23.3 (4.6)	23.5 (4.8)
**BMI SDS (WHO)**	total		0.35 (1.21)	0.50 (1.24)	0.55 (1.25)
	sports club total		0.24 (1.08)	0.36 (1.15)	0.40 (1.13)
	no sports club total		0.42 (1.28)	0.59 (1.27)	0.63 (1.29)
	girls	total	0.41 (1.16)	0.50 (1.18)	0.52 (1.23)
		sports club	0.22 (1.01)	0.26 (1.07)	0.29 (1.14)
		no sports club	0.50 (1.21)	0.61 (1.22)	0.62 (1.25)
	boys	total	0.29 (1.27)	0.50 (1.30)	0.57 (1.26)
		sports club	0.26 (1.12)	0.41 (1.19)	0.47 (1.13)
		no sports club	0.34 (1.43)	0.60 (1.41)	0.68 (1.40)
**6-min run SDS (GMT)**	total		0.15 (1.08)	ND	- 0.96 (1.01)
	sports club total		0.47 (0.98)	ND	-0.68 (0.98)
	no sports club total		-0.09 (1.10)	ND	-1.16 (0.99)
	girls	total	0.12 (1.03)	ND	-0.96 (0.98)
		sports club	0.37 (0.94)	ND	-0.80 (1.02)
		no sports club	-0.01 (1.05)	ND	-1.03 (0.96)
	boys	total	0.18 (1.13)	ND	-0.96 (1.04)
		sports club	0.53 (1.00)	ND	-0.62 (0.94)
		no sports club	-0.21 (1.15)	ND	-1.35 (1.01)

Sample size for anthropometric data, N = 764; for 6-min run test, N = 763. kg = kilogram, cm = centimetre, EQUI BMI (AUT) = equivalent BMI based on Austrian reference centile curves passing through adult BMI values (Mayer et al, 2015), WHO = BMI SDS based on World

© 2021 Jarnig G et al. *JAMA Network Open*.

Health Organization reference centile curves (de Onis et al, 2007), BMI = body mass index, SDS = standard deviation score, GMT = 6-min run SDS based on the German Motor Test (Bös et al, 2016), ND = not determined.

© 2021 Jarnig G et al. *JAMA Network Open*.

eTable 5. 3-way mixed ANOVAs for 6-min run SDS using GMT reference values

		**Effects**	**df**	**F**	***P* Value**	η_p^2	**Power**[a]
**GMT (Bös et al)**	Between-subjects effects	Gender	1	0.451	.502	.001	.103
		Sports Club	1	58.080	<.001	.071	>.99
		Gender*Sports Club	1	10.150	.002	.013	.889
		Error	759				
	Within-subjects effects	Time (T1-T3)	1	1112.765	<.001	.595	>.99
		Time*Gender	1	0.303	.582	<.001	.085
		Time*Sports Club	1	1.291	.256	.002	.206
		Time*Gender*Sports Club	1	0.918	.338	.001	.160
		Error (Time)	759				

[a] observed power computed using alpha = .05
ANOVA = analysis of variance, df = degrees of freedom, η_p^2 = partial eta square, GMT = 6-min run SDS based on the German Motor Test (Bös et al, 2016), SDS = standard deviation score.

© 2021 Jarnig G et al. *JAMA Network Open*.

eTable 6. Post-hoc tests for BMI SDS for the main effect time and time*gender interaction based on the estimated marginal means

		Pairwise comparisons	Mean diff (95% CI)	SE	p-lvl	*P* Value[a]
**IOTF (Cole et al)**	Time	T1 vs T2	-0.124 (-0.163 to -0.085)	0.016	***	<.001
		T1 vs T3	-0.163 (-0.203 to -0.123)	0.017	***	<.001
		T2 vs T3	-0.039 (-0.074 to -0.003)	0.015	*	.03
	Time*Gender (Girls)	T1 vs T2	-0.075 (-0.133 to -0.018)	0.024	**	.005
		T1 vs T3	-0.093 (-0.152 to -0.035)	0.024	***	<.001
		T2 vs T3	-0.018 (-0.070 to 0.034)	0.022		1.000
	Time*Gender (Boys)	T1 vs T2	-0.173 (-0.226 to -0.119)	0.022	***	<.001
		T1 vs T3	-0.233 (-0.287 to -0.178)	0.023	***	<.001
		T2 vs T3	-0.060 (-0.108 to -0.012)	0.020	**	.009
**AUT (Mayer et al)**	Time	T1 vs T2	-0.307 (-0.413 to -0.201)	0.044	***	<.001
		T1 vs T3	-0.428 (-0.541 to -0.314)	0.047	***	<.001
		T2 vs T3	-0.121 (-0.211 to -0.030)	0.038	**	.004
	Time*Gender (Girls)	T1 vs T2	-0.141 (-0.296 to 0.014)	0.065		.09
		T1 vs T3	-0.223 (-0.388 to -0.057)	0.069	**	.004
		T2 vs T3	-0.082 (-0.215 to 0.050)	0.055		.41
	Time*Gender (Boys)	T1 vs T2	-0.473 (-0.617 to -0.329)	0.060	***	<.001
		T1 vs T3	-0.632 (-0.786 to -0.478)	0.064	***	<.001
		T2 vs T3	-0.159 (-0.283 to -0.036)	0.051	**	.006
**WHO (de Onis et al)**	Time	T1 vs T2	-0.143 (-0.187 to -0.099)	0.018	***	<.001
		T1 vs T3	-0.190 (-0.235 to -0.144)	0.019	***	<.001
		T2 vs T3	-0.047 (-0.087 to -0.007)	0.019	*	.01
	Time*Gender (Girls)	T1 vs T2	-0.076 (-0.141 to -0.012)	0.027	*	.01
		T1 vs T3	-0.099 (-0.165 to -0.033)	0.028	***	<.001
		T2 vs T3	-0.022 (-0.081 to 0.036)	0.024		1.00
	Time*Gender (Boys)	T1 vs T2	-0.209 (-0.269 to -0.149)	0.025	***	<.001
		T1 vs T3	-0.280 (-0.342 to -0.219)	0.026	***	<.001
		T2 vs T3	-0.071 (-0.125 to -0.017)	0.023	**	.005

[a] adjusted for multiple comparisons using Bonferroni correction.
p-lvl (*P* Value level) * = *P* <.05, * = *P* <.01, * = *P* <.001, BMI = body mass index, CI = confidence interval, AUT = equivalent BMI based on Austrian reference centile curves passing through adult BMI values (Mayer et al, 2015), IOTF = BMI SDS based on International Obesity Taskforce reference centile curves (Cole et al, 2012), Mean diff = mean difference based on the estimated marginal means, p-lvl = significance level, SDS = standard deviation score, SE = standard error, T1= baseline measurements in Sept and Oct 2019, T2 = follow-up measurements in May and Jun 2020, T2 = follow-up measurements in Sep and Oct 2020, WHO = BMI SDS based on World Health Organization reference centile curves (de Onis et al, 2007).

© 2021 Jarnig G et al. *JAMA Network Open*.

eTable 7. 3-way mixed ANOVAs for BMI SDS using Austrian and WHO reference values

		**Effects**	**df**	**F**	***P* Value**	$\boldsymbol{\eta_p^2}$	**Power[a]**
**AUT (Mayer et al)**	Between-subjects effects	Gender	1	7.22	.007	.009	.77
		Sports Club	1	10.27	.001	.013	.89
		Gender*Sports Club	1	0.2	.60	<.001	.08
		Error	760				
	Within-subjects effects	Time (T1-T2-T3)	1.88	52.192	<.001	.064	>.99
		Time*Gender	1.88	12.73	<.001	.016	>.99
		Time*Sports Club	1.88	5.71	.004	.007	.85
		Time*Gender*Sports Club	1.88	2.32	.10	.003	.46
		Error (Time)	1429.15				
**WHO (de Onis et al)**	Between-subjects effects	Gender	1	0.22	.64	<.001	.08
		Sports Club	1	6.91	.009	.009	.75
		Gender*Sports Club	1	0.81	.37	.001	.15
		Error	760				
	Within-subjects effects	Time (T1-T2-T3)	1.95	60.66	<.001	.074	>.99
		Time*Gender	1.95	13.70	<.001	.018	>.99
		Time*Sports Club	1.95	3.84	.02	.005	.69
		Time*Gender*Sports Club	1.95	0.63	.53	.001	.15
		Error (Time)	1484.95				

[a] observed power computed using alpha = .05

ANOVA = analysis of variance, AUT = equivalent BMI based on Austrian reference centile curves passing through adult BMI values (Mayer et al, 2015), BMI = body mass index, df = degrees of freedom, $\boldsymbol{\eta_p^2}$ = partial eta square, SDS = standard deviation score, WHO = BMI SDS based on World Health Organization reference centile curves (de Onis et al, 2007).

© 2021 Jarnig G et al. *JAMA Network Open*.

eTable 8. Cochran's Q Test for dichotomous BMI classification using IOTF, Austrian and WHO reference values

		n	Q	df	p-lvl	*P* Value
**IOTF (Cole et al)**	total	764	18.029	2	***	<.001
	girls	383	5.143	2		.08
	boys	381	15.500	2	***	<.001
**AUT (Mayer et al)**	total	764	27.360	2	***	<.001
	girls	383	13.364	2	**	.001
	boys	381	15.643	2	***	<.001
**WHO (de Onis et al)**	total	764	29.216	2	***	<.001
	girls	383	3.526	2		.17
	boys	381	31.051	2	***	<.001

p-lvl (*P* Value level) * = *P* <.05, * = *P* <.01, * = *P* <.001, AUT = Austrian reference values using centile curves for BMI 25.00 at the age of 18 as thresholds for overweight and above (Mayer et al, 2015), BMI = body mass index, Q = Cochran`s Q test statistic, df = degree of freedom, IOTF = International Obesity Taskforce reference values using centile curves for BMI 25.00 at the age of 18 as thresholds for overweight and above (Cole et al, 2012), p-lvl = significance level, SDS = standard deviation score, WHO = World Health Organization reference values using +1 SDS percentiles as thresholds for overweight and above (de Onis et al, 2007).

© 2021 Jarnig G et al. *JAMA Network Open*.

eTable 9. Post-hoc tests for dichotomous BMI classification using IOTF, Austrian and WHO reference values

		Pairwise comparison	STS	SE	p-lvl	*P* Value[b]
**IOTF (Cole et al)**	total	T1 vs T2	2.049	0.009		.12
		T1 vs T3	4.245	0.009	***	<.001
		T2 vs T3	2.196	0.009		.08
	girls	T1 vs T2[a]				
		T1 vs T3[a]				
		T2 vs T3[a]				
	boys	T1 vs T2	1.852	0.011		.19
		T1 vs T3	3.935	0.011	***	<.001
		T2 vs T3	2.083	0.011		.11
**AUT (Mayer et al)**	total	T1 vs T2	3.118	0.008	**	.005
		T1 vs T3	5.196	0.008	***	<.001
		T2 vs T3	2.078	0.008		.11
	girls	T1 vs T2	2.872	0.010	*	.01
		T1 vs T3	3.395	0.010	**	.002
		T2 vs T3	0.522	0.010		>.99
	boys	T1 vs T2	1.620	0.011		.32
		T1 vs T3	3.935	0.011	***	<.001
		T2 vs T3	2.315	0.011		.06
**WHO (de Onis et al)**	total	T1 vs T2	4.104	0.011	***	<.001
		T1 vs T3	5.099	0.011	***	<.001
		T2 vs T3	0.995	0.011		.96
	girls	T1 vs T2[a]				
		T1 vs T3[a]				
		T2 vs T3[a]				
	boys	T1 vs T2	3.827	0.016	***	<.001
		T1 vs T3	5.421	0.016	***	<.001
		T2 vs T3	1.594	0.016		.33

[a] pairwise comparisons were not performed due to non-significant omnibus test.
[b] adjusted for multiple comparisons using Bonferroni correction.
p-lvl (*P* Value level) * = *P* <.05, * = *P* <.01, * = *P* <.001, AUT = Austrian reference values using centile curves for BMI 25.00 at the age of 18 as thresholds for overweight and above (Mayer et al, 2015), BMI = body mass index, IOTF = International Obesity Taskforce reference values using centile curves for BMI 25.00 at the age of 18 as thresholds for overweight and above (Cole et al, 2012), p-lvl = significance level, SDS = standard deviation score, SE = standard error, STS = standard test statistic, T1= baseline measurements in Sept and Oct 2019, T2 = follow-up measurements in May and Jun 2020, T3 = follow-up measurements in Sep and Oct 2020, WHO = World Health Organization reference values using +1 SDS percentiles as thresholds for overweight and above (de Onis et al, 2007).

© 2021 Jarnig G et al. *JAMA Network Open*.

eTable 10. Cochran's Q Test for dichotomous BMI classification using IOTF, Austrian and WHO reference values

<table>
<tr><th></th><th></th><th></th><th>n</th><th>Q</th><th>df</th><th>p-lvl</th><th>P Value</th></tr>
<tr><td rowspan="4">IOTF
(Cole et al)</td><td rowspan="2">girls</td><td>sports club</td><td>119</td><td>3.733</td><td>2</td><td></td><td>.16</td></tr>
<tr><td>no sports club</td><td>264</td><td>2.074</td><td>2</td><td></td><td>.36</td></tr>
<tr><td rowspan="2">boys</td><td>sports club</td><td>203</td><td>6.533</td><td>2</td><td>*</td><td>.04</td></tr>
<tr><td>no sports club</td><td>178</td><td>9.385</td><td>2</td><td>**</td><td>.009</td></tr>
<tr><td rowspan="4">AUT
(Mayer et al)</td><td rowspan="2">girls</td><td>sports club</td><td>119</td><td>7.714</td><td>2</td><td>*</td><td>.02</td></tr>
<tr><td>no sports club</td><td>264</td><td>7.600</td><td>2</td><td>*</td><td>.02</td></tr>
<tr><td rowspan="2">boys</td><td>sports club</td><td>203</td><td>4.933</td><td>2</td><td></td><td>.09</td></tr>
<tr><td>no sports club</td><td>178</td><td>11.692</td><td>2</td><td>**</td><td>.003</td></tr>
<tr><td rowspan="4">WHO
(de Onis et al)</td><td rowspan="2">girls</td><td>sports club</td><td>119</td><td>1.273</td><td>2</td><td></td><td>.53</td></tr>
<tr><td>no sports club</td><td>264</td><td>2.741</td><td>2</td><td></td><td>.25</td></tr>
<tr><td rowspan="2">boys</td><td>sports club</td><td>203</td><td>9.484</td><td>2</td><td>**</td><td>.009</td></tr>
<tr><td>no sports club</td><td>178</td><td>24.071</td><td>2</td><td>***</td><td><.001</td></tr>
</table>

p-lvl (*P* Value level) * = *P* <.05, * = *P* <.01, * = *P* <.001, AUT = Austrian reference values using centile curves for BMI 25.00 at the age of 18 as thresholds for overweight and above (Mayer et al, 2015), BMI = body mass index, Q = Cochran`s Q test statistic, df = degree of freedom, IOTF = International Obesity Taskforce reference values using centile curves for BMI 25.00 at the age of 18 as thresholds for overweight and above (Cole et al, 2012), p-lvl = significance level, SDS = standard deviation score, WHO = World Health Organization reference values using +1 SDS percentiles as thresholds for overweight and above (de Onis et al, 2007).

© 2021 Jarnig G et al. *JAMA Network Open*.

eTable 11. Post-hoc tests for dichotomous BMI classification using IOTF, Austrian and WHO reference values

			**Pairwise comparison**	**STS**	**SE**	**p-lvl**	***P* Value[b]**
**IOTF (Cole et al)**	girls	sports club	T1 vs T2 [a]				
			T1 vs T3 [a]				
			T2 vs T3 [a]				
		no sports club	T1 vs T2 [a]				
			T1 vs T3 [a]				
			T2 vs T3 [a]				
	boys	sports club	T1 vs T2	-0.949	0.016		>.99
			T1 vs T3	-2.530	0.016	*	.03
			T2 vs T3	-1.581	0.016		.34
		no sports club	T1 vs T2	-1.698	0.017		.27
			T1 vs T3	-3.057	0.017	**	.007
			T2 vs T3	-1.359	0.017		.52
**AUT (Mayer et al)**	girls	sports club	T1 vs T2	1.389	0.018		.50
			T1 vs T3	2.777	0.018	*	.02
			T2 vs T3	1.389	0.018		.50
		no sports club	T1 vs T2	2.530	0.012	*	.03
			T1 vs T3	2.214	0.012		.08
			T2 vs T3	-0.316	0.012		>.99
	boys	sports club	T1 vs T2 [a]				
			T1 vs T3 [a]				
			T2 vs T3 [a]				
		no sports club	T1 vs T2	-1.359	0.017		.52
			T1 vs T3	-3.397	0.017	**	.002
			T2 vs T3	-2.038	0.017		.13
**WHO (de Onis et al)**	girls	sports club	T1 vs T2 [a]				
			T1 vs T3 [a]				
			T2 vs T3 [a]				
		no sports club	T1 vs T2 [a]				
			T1 vs T3 [a]				
			T2 vs T3 [a]				
	boys	sports club	T1 vs T2	-2.420	0.022	*	.047
			T1 vs T3	-2.860	0.022	*	.013
			T2 vs T3	-0.440	0.022		>.99
		no sports club	T1 vs T2	-3.009	0.024	**	.008
			T1 vs T3	-4.861	0.024	***	<.001
			T2 vs T3	-1.852	0.024		.19

[a] pairwise comparisons were not performed due to non-significant omnibus test.
[b] adjusted for multiple comparisons using Bonferroni correction.
p-lvl (*P* Value level) * = *P* <.05, * = *P* <.01, * = *P* <.001, AUT = Austrian reference values using centile curves for BMI 25.00 at the age of 18 as thresholds for overweight and above (Mayer et al, 2015), BMI = body mass index, IOTF = International Obesity Taskforce reference values using centile curves for BMI 25.00 at the age of 18 as thresholds for overweight and above (Cole et al, 2012), p-lvl = significance level, SDS = standard deviation score, SE = standard error, STS = standard test statistic, T1= baseline measurements in Sept and Oct 2019, T2 = follow-up measurements in May and Jun 2020, T3 = follow-up measurements in Sep and Oct 2020, WHO = World Health Organization reference values using +1 SDS percentiles as thresholds for overweight and above (de Onis et al, 2007).

© 2021 Jarnig G et al. *JAMA Network Open*.

eTable 12. Children with overweight and obesity using Austrian and WHO reference thresholds

		September 2019 ow+ob No. (%)	June 2020 ow+ob No. (%)	September 2020 ow+ob No. (%)
**BMI categories (AUT)**	total (n=764)	114 (14.9%)	132 (17.3%)	144 (18.8%)
	sports club total (n=322)	38 (11.8%)	44 (13.7%)	51 (15.8%)
	no sports club total (n=442)	76 (17.2%)	88 (19.9%)	93 (21.0%)
	girls total (n=383)	57 (14.9 %)	68 (17.8 %)	70 (18.3 %)
	girls sports club (n=119)	10 (8.4 %)	13 (10.9 %)	16 (13.4 %)
	girls no sports club (n=264)	47 (17.8 %)	55 (20.8 %)	54 (20.5 %)
	boys total (n=381)	57 (15.0 %)	64 (16.8 %)	74 (19.4 %)
	boys sports club (n=203)	28 (13.8 %)	31 (15.3 %)	35 (17.2 %)
	boys no sports club (n=178)	29 (16.3 %)	33 (18.5 %)	39 (21.9 %)
**BMI categories (WHO)**	total (n=764)	202 (26.4%)	235 (30.8%)	243 (31.8%)
	sports club total (n=322)	76 (22.6%)	89 (27.6%)	92 (28.68%)
	no sports club total (n=442)	126 (28.5%)	146 (33.0%)	151 (34.2%)
	girls total (n=383)	112 (29.2 %)	121 (31.6 %)	119 (31.1 %)
	girls sports club (n=119)	28 (23.5 %)	30 (25.2 %)	31 (26.1 %)
	girls no sports club (n=264)	84 (31.8 %)	91 (34.5 %)	88 (33.3 %)
	boys total (n=381)	90 (23.6 %)	114 (29.9 %)	124 (32.5 %)
	boys sports club (n=203)	48 (23.6 %)	59 (29.1 %)	61 (30.0 %)
	boys no sports club (n=178)	42 (23.6 %)	55 (30.9 %)	63 (35.4 %)

AUT = Austrian reference values using centile curves for BMI 25.00 at the age of 18 as thresholds for overweight and above (Mayer et al, 2015), BMI = body mass index, SDS = standard deviation score, ow+ob = overweight including obesity, WHO = World Health Organization reference values using +1 SDS percentiles as thresholds for overweight and above (de Onis et al, 2007). No. = absolute number;

© 2021 Jarnig G et al. *JAMA Network Open*.

eFigure 1. COVID-19 restrictions in Austria between January 31, 2020 and September 30, 2020

eFigure 1A. Restriction levels for primary school children.

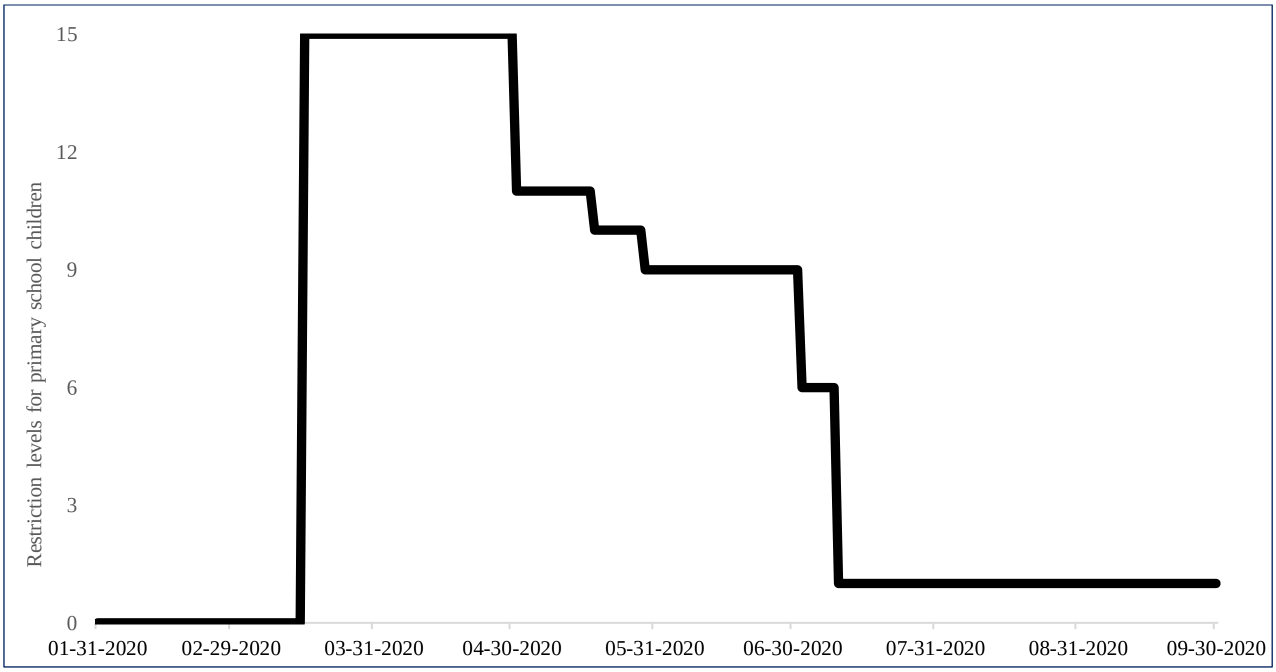

eFigure 1B. OxCGRT - Stringency Index.

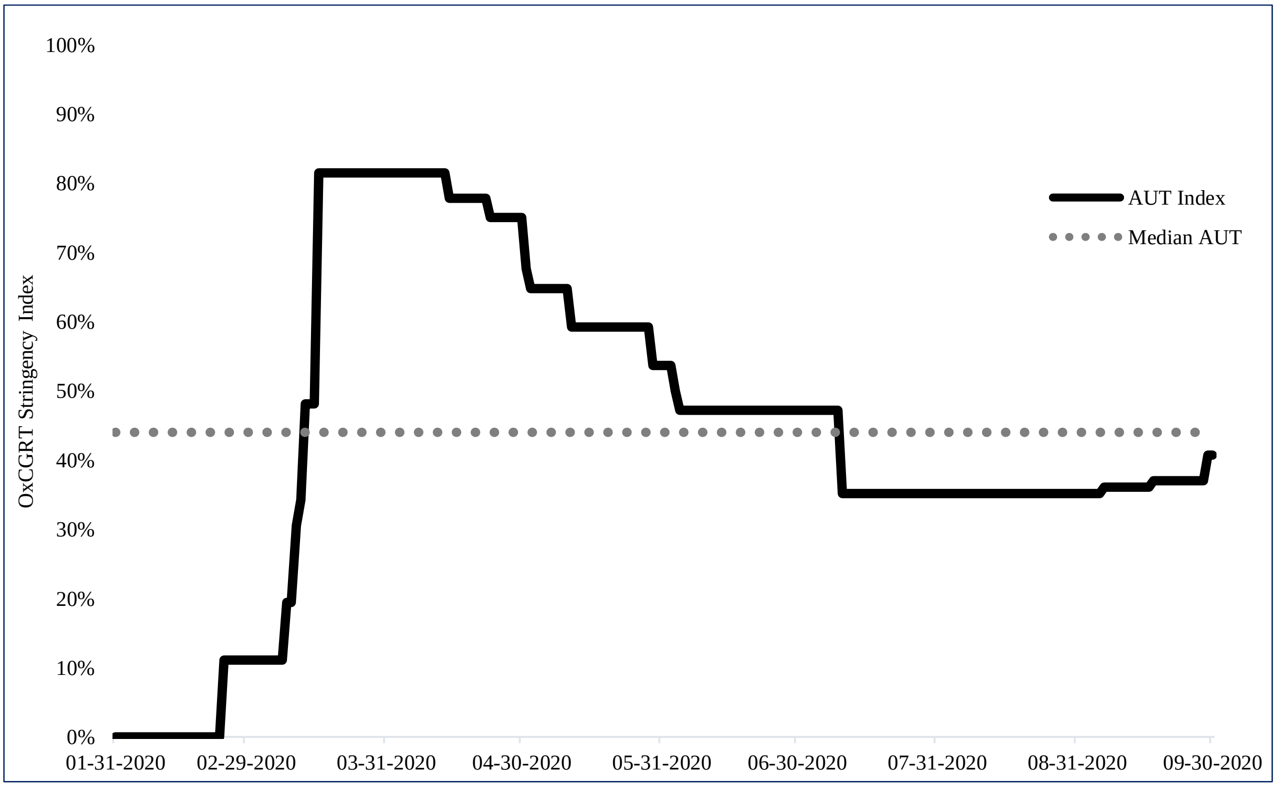

The detailed description of this classification method used in Figure 1A is available in eTable 1. Figure 1B shows the Oxford COVID-19 Government Response Tracker (OxCGRT) Stringency Index for Austria between January 31, 2020 and September 30, 2020.

© 2021 Jarnig G et al. *JAMA Network Open*.

eFigure 2. Percentage of overweight and obese children according to IOTF thresholds for girls and boys by sports club membership

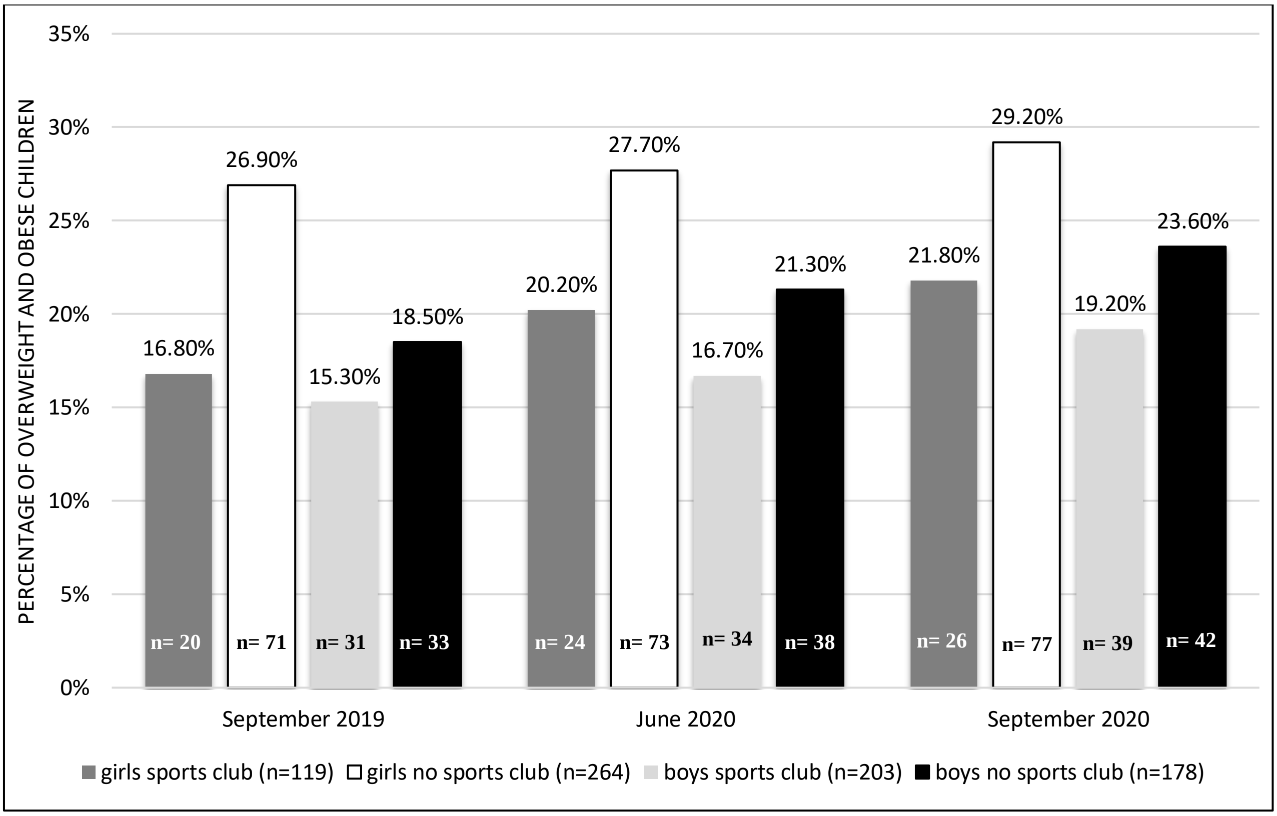

© 2021 Jarnig G et al. *JAMA Network Open*.
